# Pretreatment thrombocytosis as an independent predictive factor for chemoresistance and poor survival in epithelial ovarian cancer

**DOI:** 10.1186/s13048-020-00651-6

**Published:** 2020-05-06

**Authors:** Sari Nakao, Takeo Minaguchi, Hiroya Itagaki, Yoshihiko Hosokawa, Ayumi Shikama, Nobutaka Tasaka, Azusa Akiyama, Hiroyuki Ochi, Koji Matsumoto, Toyomi Satoh

**Affiliations:** 1 Department of Obstetrics and Gynecology, Faculty of Medicine, University of Tsukuba, 1-1-1 Tennoudai, Tsukuba, Ibaraki, 305-8575 Japan; 2 Department of Obstetrics and Gynecology, Showa University School of Medicine, Tokyo, Japan

**Keywords:** Thrombocytosis, Ovarian cancer, Survival

## Abstract

**Background:**

Thrombocytosis is related to tumor stage and survival in ovarian cancer in addition to the common complications of malignant diseases, such as anemia and inflammation. The aim of our study was to clarify the precise prognostic impact of pretreatment thrombocytosis in epithelial ovarian cancer.

**Methods:**

We retrospectively analyzed 280 consecutive patients who were treated for epithelial ovarian cancer at our institution between 2001 and 2011.

**Results:**

Pretreatment thrombocytosis was observed in 18.9% of all patients and was associated with advanced FIGO stage, primary treatment, operation achievement, histologic subtype, microcytic hypochromic anemia (MHA), and nonmalignant inflammatory condition (*P* = 0.0018, 0.0028, 0.00050, 0.034, 0.00090 and 0.0022). In the patients who relapsed after primary adjuvant chemotherapy (*n* = 126), thrombocytosis was associated with a shorter treatment-free interval (TFI) (*P* = 0.0091). The univariate and multivariate analyses revealed that thrombocytosis was independently associated with TFI and MHA (*P* = 0.021 and 0.0091). Patients with thrombocytosis had worse progression-free survival (PFS) and overall survival (OS) than those without thrombocytosis (*P* <  0.0001 and <  0.0001). The multivariate analyses for prognostic factors demonstrated that thrombocytosis was significant for poor PFS and OS (*P* = 0.0050 and 0.022) independent of stage, histology, primary treatment, operation achievement, nonmalignant inflammatory condition and MHA.

**Conclusions:**

The current findings indicate that the detrimental survival impact of pretreatment thrombocytosis in epithelial ovarian cancer may be independent of tumor extent but rather attributed to chemoresistance, further supporting the therapeutic potential of targeting thrombopoietic cytokines in the disease.

## Background

Approximately half of all patients with ovarian cancer are diagnosed with advanced-stage disease [1], as early-stage patients tend to rarely have subjective symptoms due to the anatomical location of the ovary as an intraperitoneal organ. The principal treatment for epithelial ovarian cancer is maximal cytoreduction, which typically comprises surgery followed by chemotherapy, and the amount of residual tumor is one of the most important prognostic factors [2–5]. Accordingly, the elucidation of mechanisms for tumor growth and metastasis will contribute to improving patient prognosis. Thrombocytosis is traditionally known to be associated with patient prognosis in ovarian cancer [6–15]. Platelets are involved in tumor growth, angiogenesis, and metastasis [16]. The functions of cytokines on platelet-mediated tumor proliferation and progression have been widely investigated [16]. Recently, antiplatelet therapies including molecular agents targeting thrombopoietic cytokines have been investigated by clinical trials in patients with ovarian cancer [17, 18]. However, the precise prognostic significance of paraneoplastic thrombocytosis is yet to be determined. Thrombocytosis is known to be induced by iron-deficiency anemia and nonmalignant inflammatory conditions in addition to malignant disease, and ovarian cancer patients, especially those with advanced-stage disease, may have these complications. The aim of our study was to investigate the detailed prognostic impact of thrombocytosis on ovarian cancer patients in order to elucidate the underlying mechanism and to identify the target patients who will benefit more from antiplatelet therapies.

## Methods

### Patients

We retrospectively reviewed the clinical records of a total of 280 consecutive patients who were treated for epithelial ovarian cancer at the University of Tsukuba Hospital between 2001 and 2011. The study protocol was approved by the Ethics Committee University of Tsukuba Hospital (H27–143). We excluded patients with multiple primary cancers, a past history of cancer, or hepatic disease from our study. Patients diagnosed with malignant transformation of mature cystic teratoma were also excluded. Thrombocytosis was defined as a platelet count ≥400,000/mm^3^ before treatment, which was calculated as the mean value of the initial and pretreatment examinations. For survival analyses, progression-free survival (PFS) was defined as the interval between the dates of the initial treatment and the first recurrence or progression of disease, and overall survival (OS) was defined as the interval between the dates of the initial treatment and the last follow-up. The treatment-free interval (TFI) was defined as the interval between the dates of the end of primary adjuvant chemotherapy and the first disease progression (*n* = 126). The stages were classified according to the International Federation of Gynecology and Obstetrics system (FIGO, 1988). The median follow-up period excluding patients who died was 81.4 months (range, 0.7–178). The patient demographics are summarized in Table 1.

**Table 1:** Patient characteristics.

Characteristics		No. (*n* = 280)
Age (years)	Mean ± SD	56.7 ± 11.9
Platelet counts (× 10^3^/ mm^3^)		
≥ 400		53 (18.9%)
< 400		227 (81.1%)
Hb (g/ dl)	Mean ± SD	11.8 ± 1.5
Microcytic hypochromic anemia		
Present		23 (8.2%)
Absent		259 (91.8%)
Primary treatment		
PDS		207 (73.9%)
NAC		73 (26.1%)
FIGO stage		
I		91 (32.5%)
II		39 (13.9%)
III		110 (39.3%)
IV		40 (14.3%)
Histologic subtype		
Serous		105 (37.5%)
Clear cell		83 (29.6%)
Others*		92 (32.9%)
Operation achievement		
Complete		206 (73.6%)
Optimal		56 (20.0%)
Suboptimal		18 (6.4%)
Nonmalignant inflammatory condition		
Present		15 (5.4%)
Absent		265 (94.6%)
CA125 (U/ mL)	Mean ± SD	1591 ± 3618

*Endometrioid, mucinous, undifferentiated, or mixed type. *Abbreviations*: *SD* standard deviation, *Hb* hemoglobin, *PDS* primary debulking surgery, *NAC* neoadjuvant chemotherapy, *FIGO* International Federation of Gynecology and Obstetrics

### Treatment

The basic surgical procedure for epithelial ovarian cancer consisted of total abdominal hysterectomy, bilateral salpingo-oophorectomy, omentectomy, and pelvic and para-aortic lymphadenectomy. Following primary debulking surgery (PDS), a combination of paclitaxel (175 mg/m^2^, day 1) and carboplatin (AUC = 6, day 1) (TC regimen) was administered every 3 weeks. Four cycles of TC were performed in patients with stage IA clear cell carcinoma. Six to 8 cycles were performed in patients with stage IC or higher disease. Neoadjuvant chemotherapy (NAC) followed by interval debulking surgery (IDS) was selected for patients with apparent stage III/IV disease and chemosensitive tumor histology, i.e., serous or endometrioid as estimated by CT, excessively elevated CA125 levels, and cytological findings of ascites [19]. For NAC, 4 cycles of TC were administered, and IDS was followed by an additional 4 cycles.

### Statistical analysis

Differences in proportions were evaluated by the χ^2^ test or Fisher’s exact test where appropriate. Differences in continuous variables were evaluated by the Wilcoxon rank-sum test. Logistic regression was used for the univariate and multivariate analyses of the clinicopathologic factors associated with thrombocytosis. Kaplan-Meier survival curves were generated and compared statistically by the log-rank test. The Cox proportional hazard model was used for the univariate and multivariate analyses for prognostic factors. *P*-values < 0.05 were considered statistically significant. All statistical analyses were performed using JMP11.0 software (SAS Institute, Cary, NC).

## Results

Thrombocytosis was observed in 18.9% of all patients (Table 1). We first examined the relationships between thrombocytosis and the clinicopathologic parameters. The rate of thrombocytosis significantly increased as the FIGO stage progressed: the rate was 9.9% (9/91) for stage I, 10.3% (4/39) for stage II, 23.6% (26/110) for stage III, and 35.0% (14/40) for stage IV (Table 2). Additionally, thrombocytosis was found to be significantly associated with microcytic hypochromic anemia (MHA), primary treatment (NAC vs. PDS), histologic subtype (serous, clear cell, or others), operation achievement (complete, optimal, or suboptimal resection), nonmalignant inflammatory condition, and CA125 level (Table 2).

**Table 2:** Relationships between pretreatment thrombocytosis and clinicopathologic parameters.

Parameters		Platelet counts (× 10^3^/ mm^3^)		*P*
		< 400 (*n* = 227)	≥ 400 (n = 53)	
Age (years)	Mean ± SD	56.8 ± 11.9	55.9 ± 11.9	0.52
Hb (g/ dl)	Mean ± SD	12.1 ± 1.4	10.9 ± 1.7	< 0.0001
Microcytic hypochromic anemia				0.00090
Present		12 (5.3%)	11 (20.8%)	
Absent		215 (94.7%)	42 (79.2%)	
Primary treatment				0.0028
PDS		177 (78.0%)	30 (56.6%)	
NAC		50 (22.0%)	23 (43.4%)	
FIGO stage				0.0018
I		82 (36.1%)	9 (17.0%)	
II		35 (15.4%)	4 (7.5%)	
III		84 (37.0%)	26 (49.1%)	
IV		26 (11.5%)	14 (26.4%)	
Histologic subtype				0.034
Serous		77 (33.9%)	28 (52.8%)	
Clear cell		70 (30.8%)	13 (24.5%)	
Others*		80 (35.3%)	12 (22.7%)	
Operation achievement				0.00050
Complete		178 (78.4%)	28 (52.8%)	
Optimal		36 (15.9%)	20 (37.7%)	
Suboptimal		13 (5.7%)	5 (9.5%)	
Nonmalignant inflammatory condition				0.0022
Present		7 (3.1%)	8 (15.1%)	
Absent		220 (96.9%)	45 (84.9%)	
CA125 (U/ mL)	Mean ± SD	1233 ± 3012	3127 ± 5278	< 0.0001

*Endometorioid, mucinous, undifferentiated, or mixed type. *Abbreviations*: *SD* standard deviation, *Hb* hemoglobin, *PDS* primary debulking surgery, *NAC* neoadjuvant chemotherapy, *FIGO* International Federation of Gynecology and Obstetrics, *TFI* treatment-free interval

We subsequently conducted univariate and multivariate analyses of the clinicopathologic factors associated with thrombocytosis. Among the factors significantly associated with thrombocytosis in Table 2, we selected MHA, FIGO stage, histologic subtype, operation achievement, and nonmalignant inflammatory condition as the factors to be analyzed (Table 3). To include the factor of TFI as well, we confined the analyses to the 126 patients who showed disease progression after primary adjuvant chemotherapy. Among the 4 significant factors from the univariate analysis, MHA and TFI were found to be significantly and independently associated with thrombocytosis (Table 3).

**Table 3:** Univariate and multivariate analyses of risk factors for pretreatment thrombocytosis.

Factors		Platelet counts (×10^3^/ mm^3^)		Univariate		Mutivariate	
		< 400 (*n* = 88)	≥ 400 (*n* = 38)	OR (95% CI)	*P*	OR (95% CI)	*P*
Microcytic hypochromic anemia					0.0022		0.0091
Present		4 (4.6%)	7 (18.4%)	7.56 (2.04–36.23)		6.52 (1.57–36.73)	
Absent		84 (95.4%)	31 (81.6%)	Ref		Ref	
FIGO stage					0.028		0.15
I/ II		17 (19.3%)	2 (5.3%)	Ref		Ref	
III/ IV		71 (80.7%)	36 (94.7%)	4.31 (1.15–28.12)		2.84 (0.72–18.95)	
Histologic subtype					0.061		–
Serous		47 (53.4%)	27 (71.1%)	2.41 (0.97–5.00)		–	
Others*		41 (46.6%)	11 (28.9%)	Ref		–	
Operation achievement					0.89		–
Complete, Optimal		78 (88.6%)	34 (89.5%)	Ref		–	
Suboptimal		10 (11.4%)	4 (10.5%)	0.92 (0.24–2.96)		–	
Non-malignant inflammatory condition					0.041		0.17
Present		4 (4.6%)	6 (15.8%)	3.94 (1.06–16.27)		2.67 (0.085–1.56)	
Absent		84 (95.4%)	32 (84.2%)	Ref		Ref	
TFI (months)	Mean ± SD	16.5 ± 23.03	8.30 ± 7.31	0.003 (0.00–0.30)	0.0091	0.0095 (0.00–0.75)	0.021

*Endometorioid, mucinous, undifferentiated, or mixed type. *Abbreviations*: *OR* odds ratio, *CI* confidence interval, *SD* standard deviation, *Hb* hemoglobin, *Ref* reference, *FIGO* International Federation of Gynecology and Obstetrics, *TFI* treatment-free interval

**Fig. 1:**
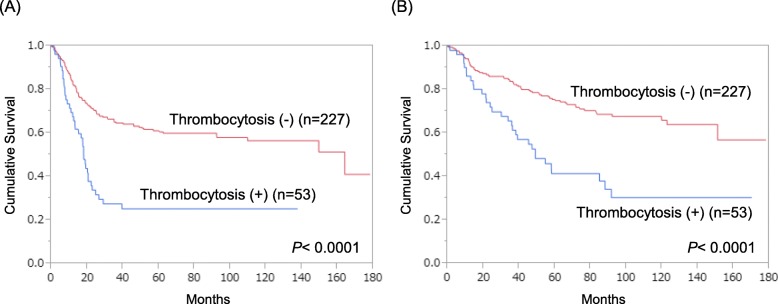
Survival curves in all patients according to the presence/absence of pretreatment thrombocytosis (*n* = 280). **a**, PFS in all patients. **b**, OS in all patients

**Fig. 2:**
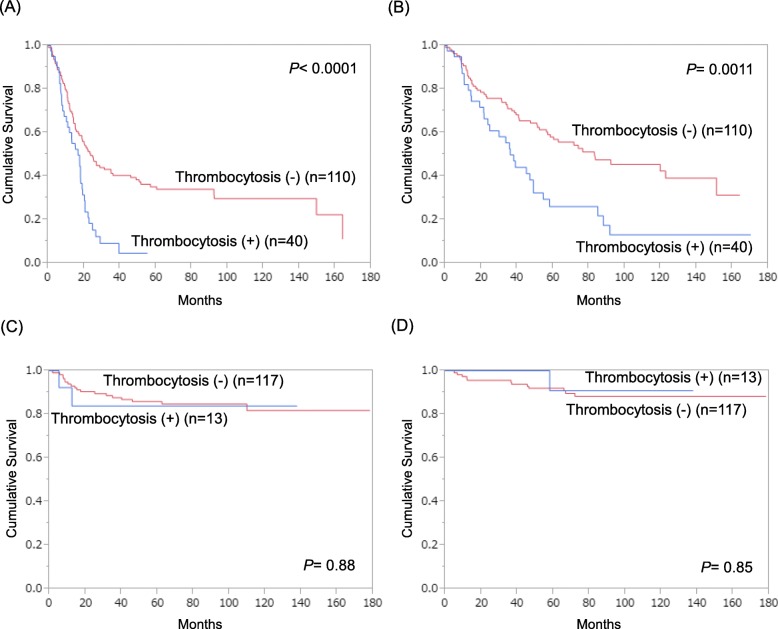
Survival curves in patients with stage III/IV (*n* = 150) or I/II (*n* = 130) disease according to the presence/absence of pretreatment thrombocytosis. **a**, PFS in stage III/IV patients. **b**, OS in stage III/IV patients. **c**, PFS in stage I/II patients. D, OS in stage I/II patients

Next, we compared PFS and OS according to the presence or absence of thrombocytosis. In all patients, those with thrombocytosis showed significantly poorer PFS (5-year PFS rate, 25.2% vs. 61.8%; Fig. 1a) and OS (5-year OS rate, 41.4% vs. 75.5%; Fig. 1b) compared to those without thrombocytosis. When the analysis was confined to the patients with stage III/IV disease, thrombocytosis was still significantly associated with poor PFS (5-year PFS rate, 0.0% vs. 34.0%; Fig. 2a) and OS (5-year OS rate, 26.1% vs. 56.9%; Fig. 2b), in contrast with the patients with stage I/II disease who showed no difference in PFS (5-year PFS rate, 86.0% vs. 83.9%; Fig. 2c) or OS (5-year OS rate, 92.0% vs. 90.1%; Fig. 2d).

Last, we performed a multivariate analysis of pretreatment thrombocytosis for OS and PFS, adjusted for age, MHA, histologic subtype, FIGO stage, primary treatment, nonmalignant inflammatory condition, and operation achievement (Table 4). Pretreatment thrombocytosis was found to be an independent prognostic factor for poor PFS and OS (Table 4).

**Table 4:** Univariate and multivariate analyses of prognostic factors for PFS and OS

	No.	PFS				OS			
		Univariate		Multivariate		Univariate		Multivariate	
Factors	(n = 280)	HR (95% CI)	*P*	Adjusted HR (95% CI)	*P*	HR (95% CI)	*P*	Adjusted HR (95% CI)	*P*
Age (years)			0.035		0.19		0.014		0.072
< 50	77	Ref		Ref		Ref		Ref	
≥ 50	203	1.53 (1.03–2.34)		1.32 (0.87–2.07)		1.80 (1.12–3.03)		1.59 (0.96–2.74)	
Platelet counts (×10^3^/mm^3^)			< 0.0001		0.0050		< 0.0001		0.022
< 400	227	Ref		Ref		Ref		Ref	
≥ 400	53	2.61 (1.76–3.80)		1.89 (1.22–2.87)		2.67 (1.72–4.06)		1.79 (1.09–2.86)	
Microcytic hypochromic anemia			0.62		0.30		0.44		0.15
Present	8	1.17 (0.59–2.07)		1.43 (0.71–2.36)		1.33 (0.62–2.49)		1.75 (0.80–3.42)	
Absent	272	Ref		Ref		Ref		Ref	
Histologic subtype			< 0.0001		0.67		< 0.0001		0.21
Serous	105	Ref		Ref		Ref		Ref	
Non-serous	175	0.36 (0.25–0.51)		0.92 (0.62–1.35)		0.45 (0.30–0.67)		1.33 (0.85–2.05)	
FIGO stage			< 0.0001		< 0.0001		< 0.0001		< 0.0001
I/ II	130	Ref		Ref		Ref		Ref	
III/ IV	150	7.80 (4.94–13.0)		5.47 (3.18–9.69)		7.92 (4.58–14.91)		6.26 (3.30–12.57)	
Primary treatment			< 0.0001		0.018		< 0.0001		0.0052
PDS	207	Ref		Ref		Ref		Ref	
NAC	73	2.99 (2.09–4.26)		1.60 (1.09–2.35)		3.29 (2.18–4.95)		1.90 (1.21–2.98)	
Nonmalignant inflammatory condition			0.061		0.55		0.0053		0.24
Absent	265	Ref		Ref		Ref		Ref	
Present	15	1.97 (0.97–3.57)		0.81 (0.38–1.56)		2.92 (1.42–5.35)		0.64 (0.33–1.37)	
Operation achievement			< 0.0001		< 0.0001		< 0.0001		< 0.0001
Complete, Optimal	262	Ref		Ref		Ref		Ref	
Suboptimal	18	7.31 (4.18–12.0)		4.41 (2.46–7.48)		6.40 (3.46–11.0)		4.60 (2.41–8.24)	

*Abbreviations*: *PFS* progression-free survival, *OS* overall survival, *HR* hazard ratio, *CI* confidence interval, *Ref* reference, *FIGO* International Federation of Gynecology and Obstetrics, *PDS* primary debulking surgery, *NAC* neoadjuvant chemotherapy

## Discussion

We observed pretreatment thrombocytosis, defined as a platelet count ≥ 400,000/mm^3^, in 18.9% of the patients with stage I-IV epithelial ovarian cancer, which is in line with previous reports using the same cutoff value as ours (7.4–42.5%) [10–12, 15]. Our analyses of the relationships between thrombocytosis and clinicopathologic parameters showed that thrombocytosis was significantly associated with MHA, primary treatment, FIGO stage, histologic subtype, operation achievement, nonmalignant inflammatory condition, CA125 level, and TFI (Tables 2 and 3). Among these significant factors, FIGO stage, CA125 level, operation achievement, and primary treatment are considered to reflect the tumor extent, which has been reportedly associated with pretreatment thrombocytosis [6, 20]. MHA and nonmalignant inflammatory condition are clinically well known to induce thrombocytosis. We subsequently conducted univariate and multivariate analyses for associations with thrombocytosis in patients who relapsed after adjuvant chemotherapy, excluding the 2 factors of CA125 level and primary treatment, which are considered to be closely related to FIGO stage. We found that MHA and TFI were significantly and independently associated with thrombocytosis (Table 3). Accordingly, thrombocytosis is suggested to possibly contribute to chemoresistance, as TFI is known to be an important surrogate marker for the chemosensitivity of ovarian cancer [21–23]. Regarding MHA, iron deficiency anemia caused by intratumoral hemorrhage in ovarian cancer is likely to be involved.

Our survival analyses showed that patients with thrombocytosis had worse PFS and OS than those without thrombocytosis (Figs. 1a, b). In addition, when the analysis was confined to stage III/IV patients, there was still a significant difference in PFS and OS (Figs. 2a, b), whereas stage I/II patients showed no difference in survival according to the presence/absence of pretreatment thrombocytosis (Figs. 2c, d). These findings indicate that thrombocytosis affects survival mainly in advanced diseases, consistent with our above finding that thrombocytosis was significantly and independently associated with TFI, an established predictor of chemosensitivity in the treatment of recurrence, as recurrence is prone to occur in advanced diseases. Furthermore, our multivariate analysis for prognostic factors demonstrated that thrombocytosis was significant for unfavorable PFS and OS independent of age, histology, and FIGO stage (Table 4). These findings indicate that pretreatment thrombocytosis may be an ideal predictive biomarker for treatment outcome and a reasonable therapeutic target in epithelial ovarian cancer.

Tumor cells first increase and activate platelets via various cytokines, including interleukin-6 (IL-6, 16). Activated platelets in turn facilitate tumor growth and angiogenesis through growth factors and angiogenic factors, including VEGF and PDGF [16, 24]. Activated platelets also promote metastasis through epithelial mesenchymal transition (EMT) and defense by platelet-tumor interactions against blood flow and the immune system, including NK cells, in circulation [16, 24]. In addition, platelets contribute to chemoresistance through MAPK and PI3-kinase/Akt pathways and drug efflux proteins [24]. Moreover, chemoresistance in ovarian cancer cells is suggested to involve the interaction between the surrounding immune system and cancer stem cells in the tumor microenvironment, where platelets play key roles [25, 26]. Therefore, thrombocytosis can possibly affect patient prognosis via both tumor progression and chemoresistance. However, we found that thrombocytosis was significantly and independently associated with TFI but not with FIGO stage (Table 3) and that thrombocytosis was significantly associated with PFS independent of FIGO stage (Table 4). These findings suggest that the prognostic impact of thrombocytosis may be independent of tumor extent but rather attributed to chemoresistance. Indeed, platelets have been reported to be involved in chemoresistance in ovarian cancer by in vitro and in vivo basic studies. Radziwon-Balicka et al. reported that platelets decreased paclitaxel-induced apoptosis in human ovarian adenocarcinoma cells in vitro [27]. Bottsford-Miller et al. reported that the combined administration of platelet-depleting antibodies and docetaxel caused a 62% decrease in tumor weight compared to docetaxel treatment in orthotopic mouse models of human ovarian cancer [6]. They further found that platelet transfusion blocked the effect of docetaxel on tumor growth, and aspirinization blocked the effect of transfusion. However, clinical evidence suggesting the link between thrombocytosis and chemoresistance in ovarian cancer is very limited, as most studies only correlate thrombocytosis with survival after chemotherapy. Bottsford-Miller et al. reported changes in platelet counts during first-line chemotherapy in responsive and refractory groups matched for stage, histology, grade, and primary therapy [28]. In patients with a durable response, only 50% had pretreatment thrombocytosis, and all of them achieved a normal platelet count during therapy, whereas all had pretreatment thrombocytosis, and only 50% achieved a normal count during therapy in patients with refractory disease. However, the possibility that platelet count only reflects the real-time residual tumor amount cannot be excluded. Feng et al. reported that preoperative thrombocytosis was significantly associated with chemoresistance determined based on the interval between disease progression and adjuvant chemotherapy in high-grade serous ovarian cancer [20]. However, thrombocytosis was not significant after stratification based on residual tumors after surgery. In our study, pretreatment thrombocytosis was not associated with operation achievement and was significantly associated with TFI independent of FIGO stage (Table 3). Moreover, pretreatment thrombocytosis was a significant prognostic factor for poor PFS and OS independent of FIGO stage and operation achievement (Table 4). These observations strongly support the involvement of thrombocytosis in chemoresistance, implicating that molecular therapy targeting thrombocytosis may improve prognosis by attenuating chemoresistance. Based on the current findings, we assume that the combination of chemotherapeutics and antiplatelet therapies may be efficacious for ovarian cancer patients with thrombocytosis. Notably, patients with MHA or nonmalignant inflammatory conditions may have to be excluded from the treatment subjects, as the pathways for thrombocytosis in these patients must be different from those for paraneoplastic thrombocytosis.

Stone et al. proposed that increased hepatic thrombopoietin synthesis in response to tumor-derived IL-6 was a mechanism for paraneoplastic thrombocytosis [29]. They further reported that treatment with siltuximab, an anti-IL-6 antibody, significantly enhanced the therapeutic efficacy of paclitaxel in mouse models of epithelial ovarian cancer. Regarding clinical trials, a phase II study in patients with platinum-resistant ovarian cancer reported that siltuximab treatment showed a partial response in one patient and disease stabilization in 7 of 18 of the evaluated patients [30]. Regarding the combination with chemotherapeutics, a phase I trial in patients with recurrent epithelial ovarian cancer reported that the combination of carboplatin/doxorubicin and tocilizumab, an anti-IL-6 receptor antibody, and interferon-α2b showed complete response in 3, partial response in 8, and stable disease in 6 of the 21 evaluated patients, and they showed that the toxicity was tolerable [31]. Additional clinical trials and the examination of clinical samples are warranted to evaluate the usefulness and to investigate the underlying mechanism of anti-IL-6 therapies in ovarian cancer.

Our study has the following limitations. First, the sample size of the subset analyses was relatively small. Second, the strengthening of our hypothesis by basic study data was lacking. Third, the retrospective study design potentially caused selection biases. Prospective studies are required to verify our findings.

## Conclusions

We reported here on the precise prognostic impact of pretreatment thrombocytosis in epithelial ovarian cancer. Univariate and multivariate analyses revealed that thrombocytosis was independently associated with TFI and MHA. Thrombocytosis was correlated with poor OS and PFS in advanced stages but showed no difference in early stages of disease. The multivariate analysis for prognostic factors demonstrated that thrombocytosis was significant for OS and PFS independent of stage, histology, primary treatment, operation achievement, nonmalignant inflammatory condition, and MHA. The current findings implicate that the unfavorable prognostic impact of thrombocytosis may be ascribed to chemoresistance, further supporting the therapeutic potential of targeting thrombopoietic cytokines in epithelial ovarian cancer.

## Data Availability

The datasets used and/or analyzed during the current study are available from the corresponding author upon reasonable request.

## Abbreviations

FIGO: International Federation of Gynecology and Obstetrics
IDS: Interval debulking surgery.
MHA: Microcytic hypochromic anemia.
NAC: Neoadjuvant chemotherapy.
OS: Overall survival.
PDS: Primary debulking surgery.
PFS: Progression-free survival.
TC: Paclitaxel and carboplatin.
TFI: Treatment-free interval.
